# Design and Evaluation of an Osteogenesis-on-a-Chip Microfluidic Device Incorporating 3D Cell Culture

**DOI:** 10.3389/fbioe.2020.557111

**Published:** 2020-09-08

**Authors:** Hossein Bahmaee, Robert Owen, Liam Boyle, Cecile M. Perrault, Andres A. Garcia-Granada, Gwendolen C. Reilly, Frederik Claeyssens

**Affiliations:** ^1^Department of Materials Science and Engineering, Kroto Research Institute, The University of Sheffield, Sheffield, United Kingdom; ^2^INSIGNEO Institute for in silico Medicine, The University of Sheffield, Sheffield, United Kingdom; ^3^Regenerative Medicine and Cellular Therapies, School of Pharmacy, University of Nottingham Biodiscovery Institute, Nottingham, United Kingdom; ^4^Eden Microfluidics, Paris, France; ^5^Grup d’Enginyeria de Productes Industrials, IQS, Universitat Ramon Llull, Barcelona, Spain

**Keywords:** organ-on-a-chip, mechanotransduction, polyHIPE, additive manufacture, bioreactor, computational fluid dynamics, tissue engineering

## Abstract

Microfluidic-based tissue-on-a-chip devices have generated significant research interest for biomedical applications, such as pharmaceutical development, as they can be used for small volume, high throughput studies on the effects of therapeutics on tissue-mimics. Tissue-on-a-chip devices are evolving from basic 2D cell cultures incorporated into microfluidic devices to complex 3D approaches, with modern designs aimed at recapitulating the dynamic and mechanical environment of the native tissue. Thus far, most tissue-on-a-chip research has concentrated on organs involved with drug uptake, metabolism and removal (e.g., lung, skin, liver, and kidney); however, models of the drug metabolite target organs will be essential to provide information on therapeutic efficacy. Here, we develop an osteogenesis-on-a-chip device that comprises a 3D environment and fluid shear stresses, both important features of bone. This inexpensive, easy-to-fabricate system based on a polymerized High Internal Phase Emulsion (polyHIPE) supports proliferation, differentiation and extracellular matrix production of human embryonic stem cell-derived mesenchymal progenitor cells (hES-MPs) over extended time periods (up to 21 days). Cells respond positively to both chemical and mechanical stimulation of osteogenesis, with an intermittent flow profile containing rest periods strongly promoting differentiation and matrix formation in comparison to static and continuous flow. Flow and shear stresses were modeled using computational fluid dynamics. Primary cilia were detectable on cells within the device channels demonstrating that this mechanosensory organelle is present in the complex 3D culture environment. In summary, this device aids the development of ‘next-generation’ tools for investigating novel therapeutics for bone in comparison with standard laboratory and animal testing.

## Introduction

One of the main impediments to drug development is the associated cost of assessing potential hazards, side effects and toxicity using pre-clinical animal testing. Clearly these are essential steps in the drug development process; however, whilst conventional *in vivo* testing approaches are viewed as the gold standard for evaluating new therapeutics they are not without shortcomings. For example, the majority of animal testing is performed in rodents despite there being a lack of similarity in the physiology and immune systems of these animals and humans. These differences are likely to be the cause of the poor translation of *in vivo* pre-clinical efficacy to human trials (McGonigle and Ruggeri, 2014; Malfait and Little, 2015).

A potential method of overcoming these limitations is using *in vitro* models to replace some facets of animal testing (Owen and Reilly, 2018). Traditionally, most *in vitro* approaches have consisted of culturing cells as a monolayer on planar, “two-dimensional (2D)” surfaces. Whilst being informative, these simple systems fail to recreate the structural and dynamic mechanical complexity of the three-dimensional (3D) environment observed within tissues and organs and therefore only provide limited information about potential tissue responses (Owen et al., 2020a).

Microfluidic cell culture platforms and “organ-on-a-chip” devices have the potential to considerably advance biological research, drug development, and resource efficiency by combining both structural and dynamic cues within *in vitro* models (Hadida and Marchat, 2019). The combination of recent advances in additive manufacturing, bioprinting and microfluidics has resulted in a surge in their development and production (Huh et al., 2011; Neuži et al., 2012). Understandably, within this research area much of the focus has been on creating models of organs involved in drug metabolism and clearance, for example liver and kidney models (Chao et al., 2009; Novik et al., 2010; Ferrell et al., 2012). However, in the future it will be crucial to also have models of target organs for the therapeutics of interest. Within this area, some microfluidic models of other organs, such as lung (Hinderer et al., 2012; Huh et al., 2012), gastrointestinal (GI) tract (Pusch et al., 2011; Esch et al., 2012), skin (Brauchle et al., 2013; Wagner et al., 2013), bone marrow (Torisawa et al., 2014; Sieber et al., 2018), and bone (Bersini et al., 2014; Hao et al., 2018), have been researched. A recent review by Scheinpflug et al. (2018), examines microfluidic, organ-on-a-chip approaches to modeling different aspects of bone *in vitro*. The ultimate hope is that by connecting different organ models together within one human-on-a-chip framework, the opportunity to study the effects of drugs and their metabolites on multiple organs will arise (Maschmeyer et al., 2015) with the potential to accelerate drug development significantly (Esch et al., 2014).

Traditional microfluidic devices are a clear improvement in physiological relevance when compared to traditional static cell culture due to the presence of fluid flow and fluid shear stress (FSS). However, despite these advances, many microfluidic devices remain “2D” at the cell-scale as cells are still ultimately cultured on a planar surface. This lack of a 3D microenvironment results in cells having a different morphology, polarity, and behavior to that observed in 3D cell culture and *in vivo* (Baker and Chen, 2012). Fortunately, advances within the parallel field of tissue engineering have revealed a range of approaches that can be used to generate 3D tissues *in vitro*, the principles of which can be adapted for and incorporated within microfluidic tissue-mimics.

Bone is a prime example of a tissue that requires both a 3D microenvironment and fluid flow for an accurate *in vitro* model to be produced. The field of bone tissue engineering allows us to address the structural problem by combining the culture of osteoprogenitor cells with scaffolding materials that provide a template for cell growth and the formation of bone-like extracellular matrix and specialized media that provide the essential chemical cues for bone development. Many different materials have been used for bone tissue engineering, ranging from metals and ceramics to natural and synthetic polymers (Bose et al., 2012; Roseti et al., 2017; Owen and Reilly, 2018). However, of notable recent interest is the development of polymerized high internal phase emulsion (polyHIPE) materials as polymeric scaffolds for bone tissue engineering (Robinson et al., 2016; Lee et al., 2017; Paterson et al., 2018; Aldemir Dikici et al., 2019; Aldemir Dikici and Claeyssens, 2020; Owen et al., 2020b). PolyHIPEs are inherently porous materials that are formed by emulsion templating; the emulsion consists of a curable continuous phase that encapsulates an immiscible internal droplet phase where the internal phase volume exceeds 74%. The continuous phase is then polymerized to produce a highly porous material with extensive pore interconnectivity where the percentage porosity is simply the internal phase volume ratio. Our previous work has demonstrated that by making the continuous phase photocurable, it is possible to additively manufacture polyHIPEs (Owen et al., 2015, 2016; Malayeri et al., 2016; Wang et al., 2016; Sherborne et al., 2018).

In this study, we detail for the first time the design, development, and assessment of a microfluidic osteogenesis-on-a-chip device that incorporates a reproducible 3D polymer scaffold and physiologically relevant flow conditions. The design was composed of two separate parts; a photocurable polyHIPE scaffold that is embedded within a silicone-based polymer microfluidic unit. To assess device efficacy, human embryonic stem cell-derived mesenchymal progenitor cells (hES-MPs) were cultured for up to 3 weeks. Different flow rates and flow profiles were examined, comparing metabolic activity, osteogenic differentiation, and mineralized matrix deposition.

## Results and Discussion

### Bone Microfluidic Chip Design

**FIGURE 1:**
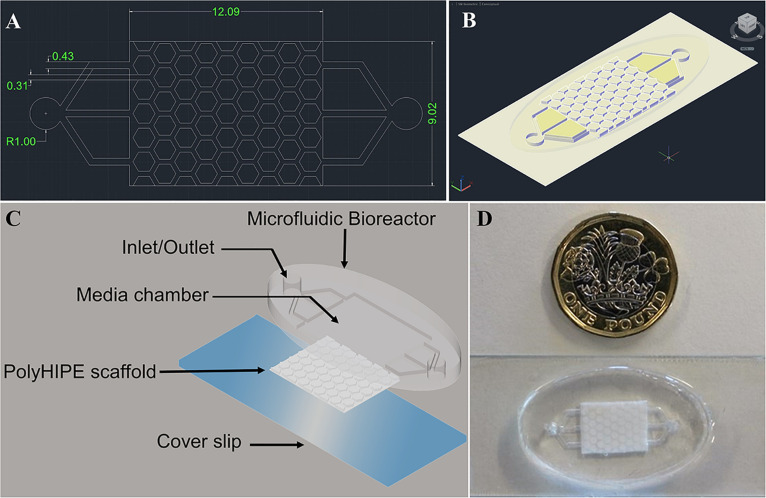
AutoCAD design of the bone microfluidic chip **(A)** 2-D and **(B)** 3D view of the combined bioreactor and polyHIPE scaffold. Dimensions are in mm. **(C)** Schematic/exploded view of the chip components, including the microfluidic bioreactor, polyHIPE scaffold and a cover slip. **(D)** Photograph of the assembled chip with a £1 (GBP) coin for scale.

We set out to design a bone microfluidic chip that fulfilled the requirements of being suitable for osteoblast culture by incorporating a 3D bone tissue engineering scaffold and physiologically relevant flow conditions, as well as being simple to manufacture *en masse*, reproducible, and cost effective. To achieve this, a two-part device consisting of a bioreactor and scaffold was designed where each component could quickly be reproducibly and easily fabricated through reusable molds (Figures 1A,B).

The bioreactor has two manifolds to act as an inlet and outlet, each consisting of a 2 mm diameter main channel and three 430 μm sub-channels which lead to the scaffold chamber. These channels are responsible for transporting media into and out of the microfluidic chip. The scaffold has a uniform repeating pattern of regular, congruent hexagonal pillars 280 μm in height with a side length of 690 μm protruding from a base 220 μm deep (total height 500 μm). Each pillar is separated by a 310 μm channel to allow regular flow through the chip. A hexagonal rather than circular pillar design was adopted to ensure that the channel width was the same throughout. From our computational fluid dynamics (CFD) assessment we observed that this reduces the variation in pressure and shear stress (see Supplementary Information).

### Bone Microfluidic Chip Fabrication and Assembly

The molds for both the bioreactor and scaffold component were fabricated from polyethylene glycol diacrylate (PEG-DA) using stereolithography in order to exercise precise control over the final architecture in comparison to other additive manufacturing techniques such as extrusion printing (Melchels et al., 2010).

The bioreactor component of the bone microfluidic chip was formed using negative replica molding with the final polydimethylsiloxane (PDMS) part produced directly from the PEG-DA mold. PDMS is a popular material for fabricating microfluidic devices due to its biocompatibility, oxygen permeability, transparency, and ease of molding into micron-scale features (Zhou et al., 2010), and it was for these reasons it was selected for the bioreactor component. Despite its prevalence in the field of microfluidics, it is worth noting that it is not the ‘perfect’ material as it readily absorbs organic solvents, biological molecules and drugs (Mukhopadhyay, 2007). Therefore, whilst the proof-of-concept device described here relies on PDMS to contain the device, further optimization could identify an alternative material for the bioreactor component to alleviate this problem.

The scaffold component of the chip was fabricated from a polyHIPE using a two-step molding process where a PDMS negative was first produced from a PEG-DA mold, and this was subsequently used to cast the polyHIPE. To assemble the bone microfluidic chip, a plasma-treated polyHIPE scaffold was inserted into the chamber of the PDMS bioreactor and this unit covalently bound onto a glass slide using air plasma to seal the microfluidic chip (Figures 1C,D).

The polyHIPE material was selected to fabricate the scaffold housed within the bioreactor as over the last decade emulsion templating has been demonstrated to be an excellent way of producing 3D scaffolds for cell culture, exemplified by the commercialization of Alvetex^®^ (Knight et al., 2011). Recent publications have structured the polyHIPE used here into complex 3D architectures using stereolithography to create scaffolds for bone tissue engineering (Owen et al., 2016; Sherborne et al., 2018). However, direct laser writing of these emulsions is relatively slow when compared to other scaffold manufacturing techniques. The comparative simplicity of the scaffold produced here made it possible to accurately produce this structure by casting the polyHIPE rather than individually printing each scaffold, an approach which increases device manufacture throughput.

### PolyHIPE Morphology Is Not Affected by Mold Stamping

**FIGURE 2:**
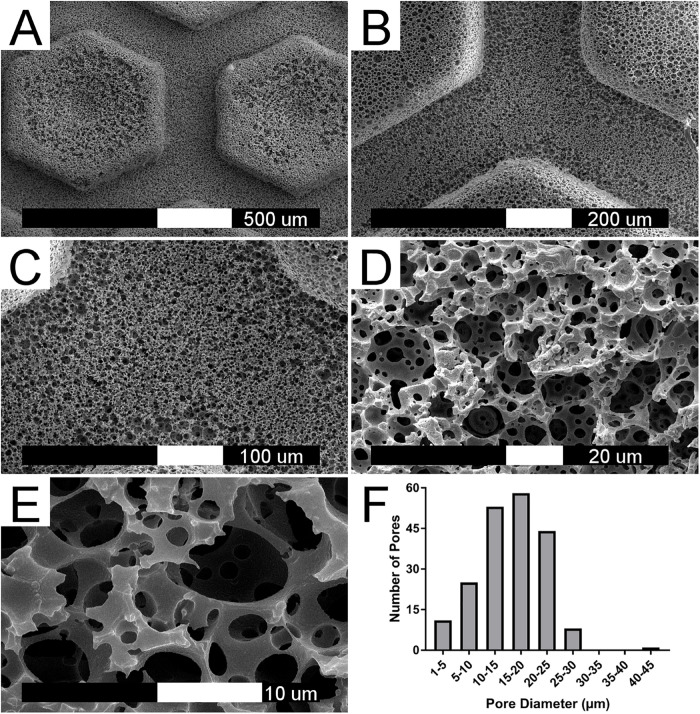
PolyHIPE scaffold morphology. **(A–E)** SEM image of 80% porosity polyHIPE scaffold at increasing magnifications. **(F)** Histogram of pore diameter distribution based on eight different SEM image analysis (*n* = 200).

Physical characterization of the polyHIPE scaffold was performed using scanning electron microscopy (SEM) (Figures 2A–E). The classical polyHIPE morphology was observed within the scaffold, demonstrating that the stamping process produced an open surface porosity with no adverse effects such as surface skin formation, which can occur during other fabrication methods (Kimmins and Cameron, 2011; Sherborne et al., 2018). Pore size distribution was analyzed using eight different SEM images taken from different scaffolds using the measurement tool within ImageJ (Schneider et al., 2012) (Figure 2F). Since the pore bisection is not always in the equatorial plane of the pore, a statistical correction factor was applied to take this underestimation into account and calculate a more accurate diameter (Barbetta and Cameron, 2004; Owen et al., 2016). The inherent polyHIPE pore sizes ranged from 5 to 30 μm, a scale suitable for cell attachment, proliferation, ingrowth and nutrient transfer. The average pore diameter within the polyHIPE scaffold was 15.90 ± 6.11 μm.

It is feasible that a monolith of the polyHIPE could have been used over the more complex channel design as flow through the scaffold would still have been achievable due to the high level of interconnected porosity. However, a design which incorporated regular channels was chosen for several reasons. First, in a monolithic design only smaller, cell-sized pores would be present which may result in blockage as extracellular matrix is produced. This would limit nutrient and waste transfer and subsequently the maximum possible duration of culture. Therefore, we hypothesized that the inclusion of channels would permit even distribution of flow within the scaffolds for longer durations before they become filled with matrix, maximizing culture duration. Second, the presence of channels increases the achievable flow velocity and subsequent shear stress which can be applied within the chip. Finally, forming channels within the polyHIPE introduces a multiscale, micro- to macro-scale porosity where macropores (the channels) are present between the microporous pillars and base of the scaffold. This hierarchical porosity is observed in native bone tissue (Genthial et al., 2017) and has been demonstrated to be beneficial for osteoblast activity (Owen et al., 2016; Sherborne et al., 2018).

Previously, microfluidic devices which incorporate a 3D architecture have been developed for study of vascularization of bone, hematopoiesis and cancer metastasis in bone (Bersini et al., 2014; Torisawa et al., 2014; Hao et al., 2018; Scheinpflug et al., 2018; Sieber et al., 2018); however, these rely on the use of random architecture ceramic scaffolds, deposited extracellular matrix, or collagen gels to provide the required 3D microenvironment. Furthermore, where the use of human or animal components in the creation of the 3D environment, availability is limited, standardization is difficult, and it is not a methodology amenable to truly replacing animal testing (Scheinpflug et al., 2018). Therefore, whilst these approaches do provide the geometric cues required to mimic native bone, they lack the inter-chip reproducibility required for systematic drug studies. Despite the precise micropore position within the polyHIPE being non-specified as it is dictated by water droplet position at the time of polymerization, the overall porosity (total porosity, pore distribution, pore size) of the material is highly consistent between samples and batches of the material, as evidenced by the small standard deviation of the pore size analysis within this study and the degree of openness characterization performed by Owen et al. (2016). Therefore, the reproducibility of the whole scaffold used within the chip remains high; an important requirement for controlled pre-clinical evaluation of pharmaceuticals.

### Higher Flow Rates Compromise Initial Cell Attachment but Not Long-Term Growth

Fluid shear stress is exerted *in vivo* when bone is deformed. It is thought to be sensed by cells through mechanoreceptors such as the primary cilia (Delaine-Smith and Reilly, 2012), which in turn promote factors that stimulate osteoblast differentiation and bone formation (Johnson et al., 1996; Sikavitsas et al., 2003; Wu et al., 2006; Temiyasathit and Jacobs, 2010). It has been previously demonstrated that when FSS is applied *in vitro*, osteogenic differentiation is promoted and limitations on nutrient transfer in larger constructs is mitigated. Efficacy of *in vitro* mechanical stimulation is sensitive to several parameters, such as magnitude of the stimulation, number of cycles and frequency. Although bone is typically loaded *in vivo* in a cyclic manner, *in vitro* there are contradicting reports about whether oscillating flow profiles are superior to other flow modalities at promoting osteogenesis, with studies finding it both better and no different to unidirectional flow (Du et al., 2009; Case et al., 2011).

**FIGURE 3:**
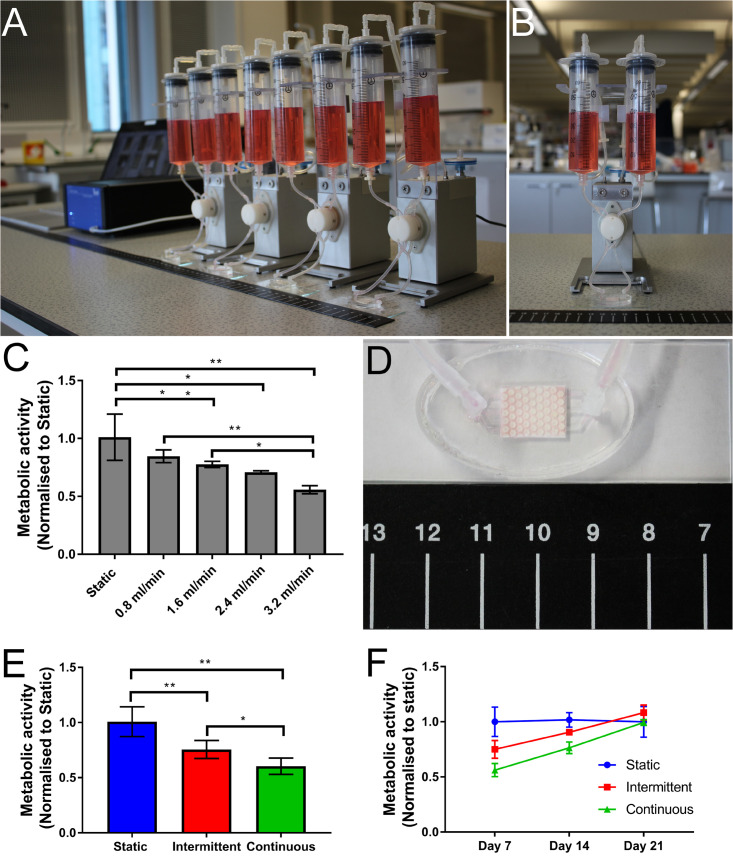
Photographs of **(A)** the Ibidi fluidic system and **(B)** a single fluidic unit. **(C)** Cell metabolic activity on day 7 normalized to static control for four different flow rates (0.8, 1.6, 2.4, and 3.2 mL/min). As flow rate increases, metabolic activity decreases (*p < 0.05, **p < 0.01) (*n* = 4). **(D)** Photograph showing a perfused bone microfluidic chip connected to the commercial Ibidi perfusion pump system with ruler for scale. **(E,F)** Comparison of metabolic activity over time with different flow profiles. **(E)** Day 7 metabolic activity. Both the intermittent (0.8–3.2 mL/min) and continuous (3.2 mL/min) flow profiles have significantly lower metabolic activity after 1 week than the static control, although the intermittent is significantly higher than the continuous. **(F)** By day 21 the cell metabolic activity has recovered for both intermittent and continuous flow profiles, resulting in no significant difference. Recovery is achieved sooner in the intermittent condition (*n* = 8) (*p < 0.05, **p < 0.01).

To assess the suitability of the bone microfluidic chip for cell growth and differentiation, hES-MPs were cultured for up to 3 weeks inside the chip in osteogenesis induction media (OIM). To identify the optimum flow profile, different flow rates and patterns were examined. Initially, four different continuous unidirectional flow rates (0.8, 1.6, 2.4, and 3.2 mL/min) were compared to a static control over a 7-days period with metabolic activity assessed on day 7 (Figure 3).

As the flow rate increased, cell metabolic activity decreased, with significantly lower metabolic activity in comparison to static at flow rates above 1.6 mL/min (Figure 3C). At the highest flow rate, cell metabolic activity after 7 days is almost 50% of that of the static control. This reduction is most likely due to the detachment of poorly attached cells at the onset of fluid flow, an effect exacerbated by higher flow rates (Cartmell et al., 2003; Jaasma and O’brien, 2008).

When applying fluid flow *in vitro*, it has been reported that the inclusion of rest periods between flow sessions enhances the effects of FSS. It is believed these intermittent flow profiles allow a ‘reset’ of the signaling pathways, stopping them from becoming saturated and allowing reactivation when flow resumes (Robling et al., 2000, 2001; Donahue et al., 2003; Batra et al., 2005; Srinivasan et al., 2007; Plunkett et al., 2009; Case et al., 2011; Gong et al., 2014). When initially exposed, both continuous and intermittent flow profiles will have the same effects as the FSS is mechanotransduced. However, under continuous flow this stimulation is subject to ‘diminishing returns,’ whereas under intermittent flow where the cells are allowed to recover the subsequent bouts of stimulation are as effective as the initial treatment (Robling and Turner, 2009).

To assess whether the initial disruption of cell metabolic activity by the 3.2 mL/min flow rate would impact the long-term performance of the chip, cultures were extended to 14 and 21 days, examining metabolic activity at each time point. To test whether the effects of a continuous high flow rate could be alleviated by rest periods, an intermittent flow regime was also investigated (Figures 3E,F). Previously, it has been found *in vivo* that rest periods of 4 to 8 h are required to regain mechanosensitivity of osteoblasts (Robling et al., 2001) and flow is typically applied for bouts of up to 2 h (Michael Delaine-Smith et al., 2015). Therefore, in this study the bone microfluidic chip was subjected to the high flow condition (3.2 mL/min) for 90 min followed by lowest flow condition (0.8 mL/min) for 270 min (4.5 h) in a repeating cycle for the duration of the experiment.

A low flow rate (0.8 mL/min) was selected for the rest period rather than zero flow as cell death was observed inside the sealed bone microfluidic chips when a flow rate of 0 mL/min was used (see Supplementary Information). It has been hypothesized that *in vivo*, bone may be continuously mechanically stimulated even whilst there is no skeletal movement due to blood pressure-induced interstitial fluid flow in the lacuno-canalicular system (Fritton and Weinbaum, 2009). However, computational models have estimated that these flow rates and subsequent shear stresses are less than 3% of the fluid flow that is induced by mechanical loading, and therefore are insufficient to promote osteogenic differentiation (Wang et al., 2003; Li et al., 2010). Therefore, the low flow rate used during the rest period of the intermittent flow profile in this study could be considered more analogous to the *in vivo* condition than a zero flow rate.

By day 7, cell metabolic activity was significantly lower in the intermittent and continuous flow profiles than the static control (Figure 3E), although the intermittent profile had significantly higher metabolic activity than the continuous indicating the benefits of rest periods. Despite this, by day 21 there was no significant difference in metabolic activity between any of the conditions, indicating that the initially lower cell metabolic activity is recovered over time (Figure 3F). Although the intermittent flow profile recovered to the levels of the static control before the continuously flowed samples, this appears to be due to a smaller initial loss of cells when flow was first applied rather than a faster recovery rate as the gradients of the intermittent and continuous profiles are comparable.

### An Intermittent Flow Profile Promotes Differentiation and Enhances Mineralized Matrix Deposition

In order to examine whether either the intermittent or continuous flow profile has any effect on osteogenic differentiation and mineralized matrix production, bone microfluidic chips were maintained in OIM for up to 21 days with either the static, intermittent (0.8–3.2 mL/min) or continuous (3.2 mL/min) flow profile. ALP activity and total DNA were quantified on day 14 and calcium deposition and collagen production on day 21.

**FIGURE 4:**
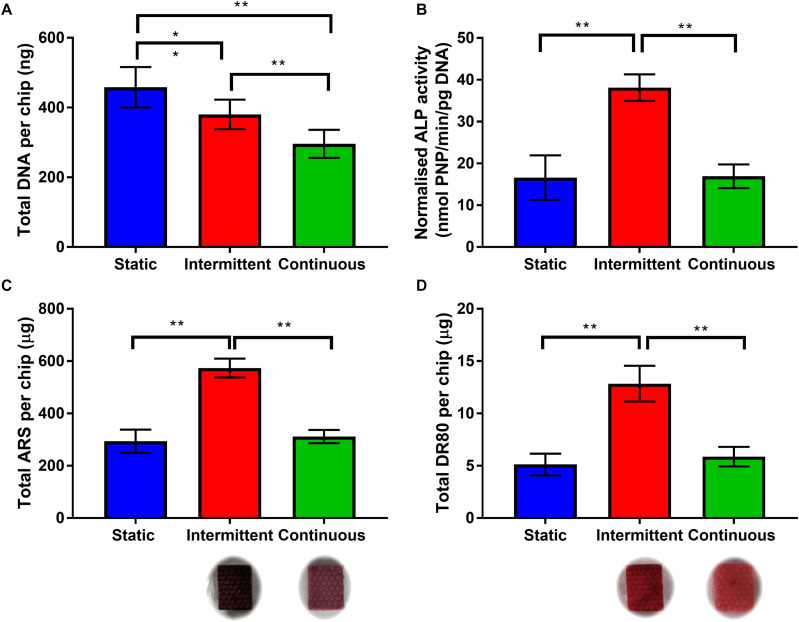
**(A)** Total DNA per chip for each flow profile on day 14. Both intermittent and continuous are significantly lower than static, with continuous significantly lower than intermittent (*n* = 8) (^∗^*p* < 0.05, ^∗∗^*p* < 0.01). **(B)** Day 14 ALP activity normalized to total DNA. The intermittent flow profile resulted in significantly higher normalized activity than the static control and continuous flow profile. There is no significant different between static and continuous. **(C,D)** Comparison of matrix deposition of hES-MPs in three different flow conditions at day 21 in OIM. **(C)** Total Alizarin Red S (ARS) staining per chip, calcium content is significantly higher for intermittent flow, there is no difference between static and continuous. **(D)** Total Direct Red 80 (DR80) staining per chip, collagen deposition is also significantly higher for intermittent flow, no difference between static and continuous. Representative images inserted below the intermittent and continuous flow conditions (*n* = 8) (**p < 0.01).

Total DNA agreed with the metabolic activity data that cell number is indeed lower when cells were exposed to flow (Figure 4A). When an intermittent unidirectional flow profile was applied, ALP activity, calcium deposition and collagen synthesis were 2.3, 1.8, and 2.2 times higher than the continuous flow profile, respectively (Figures 4B–D). Interestingly, for both ALP activity and matrix deposition, there was no significant difference between the static condition and continuous flow profile. These data demonstrate that the shear stresses the cells are exposed to during the 3.2 mL/min flow rate have the potential to promote osteogenic differentiation, but only when combined with intermittent rest periods. This agrees with previous work which found that rest periods in mechanical stimulation protocols significantly promoted osteogenesis of progenitor cells in comparison to continuous flow (Robling et al., 2000; Batra et al., 2005; Srinivasan et al., 2007; Kreke et al., 2008; Case et al., 2011). It is worth noting that in this study only unidirectional continuous and intermittent flow profiles were compared. However, future work on this system should include optimization of the flow rate and rest interval, and also compare oscillating flow as bone is typically loaded cyclically *in vivo* during normal gait.

### Cells Penetrate the PolyHIPE Network and Fill the Channels Within the Chip

**FIGURE 5:**
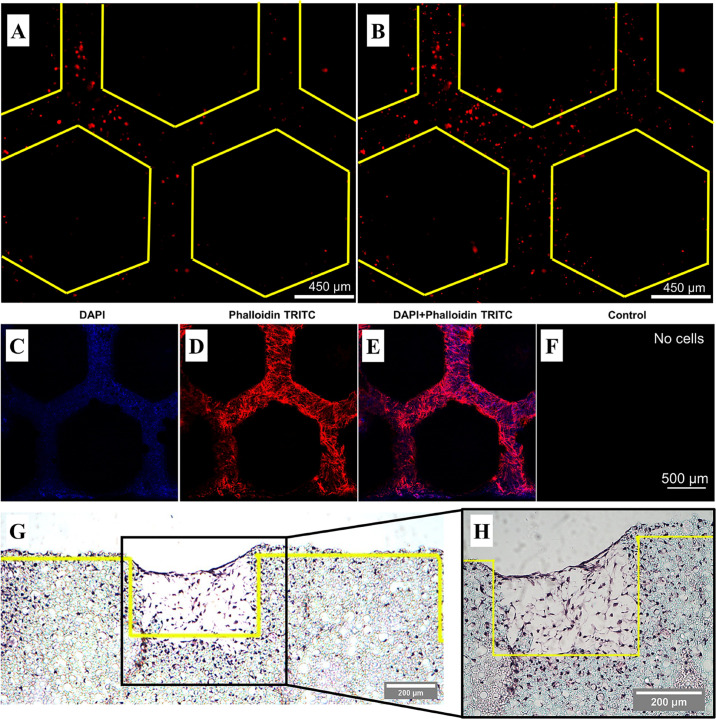
Representative fluorescence and confocal microscopy and histological sections of hES-MPs seeded in the bone microfluidic chip in OIM with the intermittent flow profile. Yellow lines demarcate the edge of the pillars. Live CellTracker^TM^ images on **(A)** day 1 and **(B)** day 3 taken through the PDMS chamber. **(C–F)** Day 14 confocal images of nuclei stained with DAPI (blue) and actin with phalloidin-TRITC (red). Individual color channels and composite shown. The control was not autofluorescent, hence it appears black. Cells are present throughout the channels and pillar walls. **(G)** Low magnification and **(H)** high magnification 8 μm histology section stained with H&E stain. Cells can be seen throughout the channels to the height of the pillars, as well as within the interconnected porous network of the polyHIPE bulk material.

*In situ* monitoring of cells was possible due to the transparency of the PDMS bioreactor component. To determine how cells behave within the microfluidic unit, hES-MPs were stained with CellTracker^TM^ and cultured for 14 days in OIM with the intermittent flow profile. Live cells were imaged on days 1 (Figure 5A) and 3 (Figure 5B) with fluorescence microscopy before fixing and examining with confocal microscopy and histology on day 14 (Figures 5C–H).

The presence of cells within the channels could easily be detected at early timepoints by staining cells with CellTracker^TM^ prior to seeding, although subsequent cell divisions result in an attenuated signal over time. Confocal microscopy revealed cells throughout the channels within the polyHIPE scaffold through the staining of nuclei (blue) and the cytoskeleton (red). Histology allowed a cross-sectional view of the scaffold, revealing that not only are cells present within the channels, but also within the interconnected porous network of the bulk polyHIPE material, indicating full penetration of the material. These could not be observed using confocal microscopy due to the light scattering nature of the polyHIPE limiting infiltration into the material. Despite the initially lower cell attachment in comparison to static of the intermittent flow profile, it is clear that significant quantities of extracellular matrix was still deposited within the channels of the scaffold.

### Fluid Shear Stress Alone Is Not Enough to Induce Osteogenic Differentiation

**FIGURE 6:**
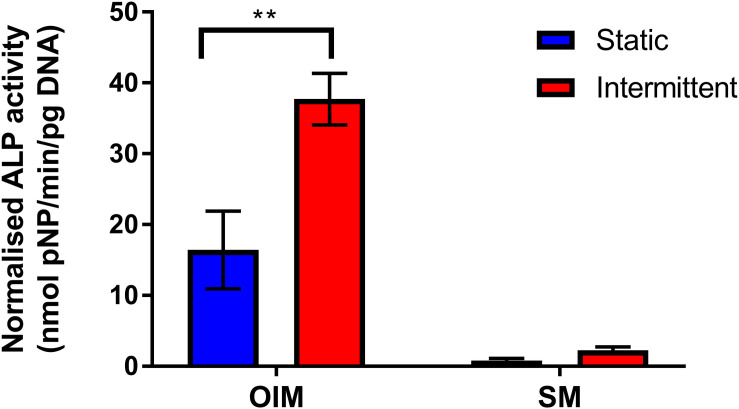
Comparison of day 14 normalized ALP activity of bone microfluidic chips maintained with the intermittent flow profile in either OIM or SM. As with Figure 4, ALP activity was significantly higher with intermittent flow on OIM. However, there was minimal ALP activity in chips maintained in SM, regardless of flow profile (*n* = 4) (**p < 0.01).

Due to the potent nature of the intermittent flow profile, it was investigated whether mechanical stimulation alone is sufficient to induce osteogenic differentiation of hES-MPs. Chips were maintained in either OIM or supplemented media (SM, OIM without dexamethasone) for 14 days with the intermittent flow profile (Figure 6). The difference between these media is that OIM contains dexamethasone, a glucocorticoid that is a powerful promoter of osteogenic differentiation. Both media contained βGP and AA2P, supplements required for matrix mineralization and collagen synthesis, respectively.

As in Figure 4, normalized ALP activity was significantly higher when chips were maintained in OIM for 14 days with the intermittent flow profile. However, ALP activity was significantly lower in SM than OIM regardless of flow profile, with no significant effect of intermittent flow on ALP activity in comparison to the static control. This is in agreement with other studies examining the effect of FSS on hES-MPs (Delaine-Smith et al., 2012; Puwanun et al., 2018), demonstrating that FSS may enhance differentiation and bone formation, but alone is not sufficient to induce osteogenic differentiation.

### Primary Cilia Are Present Within the Bone Microfluidic Chip

One mechanism by which FSS is thought to be transduced by cells is via the primary cilium. To identify whether this mechanosensor could be detected within the chip, cultures were examined after 7 days by light sheet microscopy with antibody staining for this mechanosensory organelle. Day 7 was selected as primary cilia only begin to form once cells have stopped undergoing mitosis, meaning that there are fewer to detect during the growth phase in the first week of culture.

**FIGURE 7:**
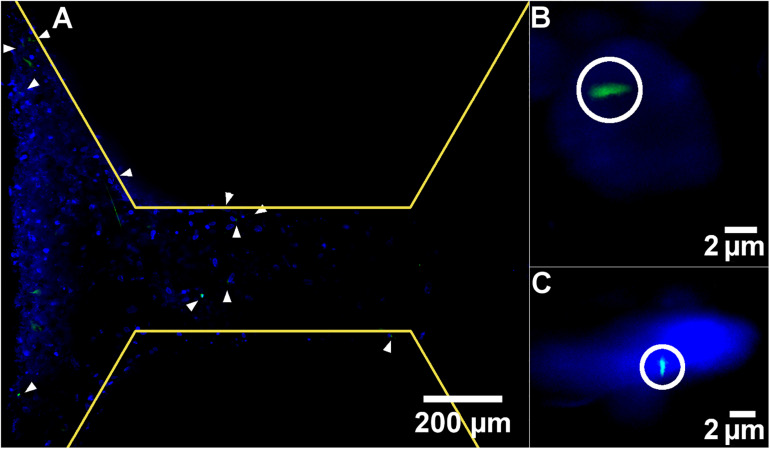
Representative light-sheet images at **(A)** low and **(B,C)** high magnification of nuclei stained with DAPI (blue) and primary cilia stained with anti-acetylated alpha tubulin (green). White arrow heads and circles indicate primary cilia. Yellow lines demarcate the edge of the pillars.

Primary cilia were found to be present on cells in the channels, indicating they would be exposed to fluid flow through the chip and are a potential mechanism by which the applied FSS could be mechanotransduced within the device (Figure 7). Although this was the only location where primary cilia were visible, this does not mean that they are not present on cells within the bulk of the polyHIPE, only that they could not be visualized here. Due to the autofloresence and light scattering nature of the polyHIPE, fluorescent imaging of such a small organelle within the polyHIPE network *in situ* was not possible. However, as they develop on the majority of quiescent mammalian cells including osteoblast-lineage cells, and are visible elsewhere in the same culture, it is highly likely they are also present on cells within the polyHIPE.

The appearance of the majority of cells on the left side of the image is not due to a difference in cell density, but because of the imaging modality. The lightsheet generated during this microscopy technique is not perfectly planar, it is thicker at the edges, meaning that a greater signal is generated here and a thicker *z* slice. It would be interesting to assess whether the different flow profiles examined here had any effect on the orientation or length of the cilia. However, quantitative measurement of cilia is notoriously difficult in 3D cell culture in general, and impossible in this study as the working distance of the lenses required to achieve sufficient magnification have a working distance shorter than the channel depth of the scaffold, meaning the cells cannot be focused upon.

### Computational Fluid Dynamics Estimates of Flow Parameters

**FIGURE 8:**
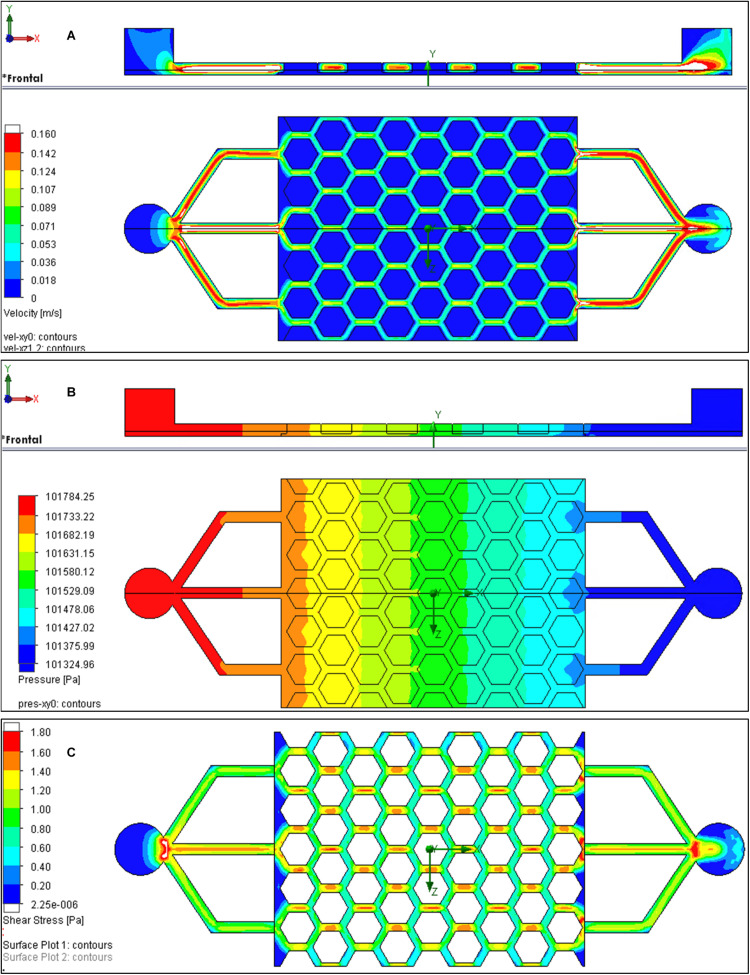
Computational fluid dynamics (CFD) results for the bone microfluidic chip at inlet flow rate of 3.2 mL/min. **(A)** Velocity and **(B)** pressure as media passes through the chip (left to right). **(C)** Shear stresses at the bottom of the channels if the polyHIPE is assumed to be non-porous.

Computational fluid dynamics was used to approximate the fluid velocity and FSS distribution within the bone microfluidic chip during the high flow period of the intermittent flow profile (Figure 8). Flow through the bulk polyHIPE material was determined experimentally via a pressure drop experiment through a defined thickness disk of the polyHIPE. The pressure drop was then simulated, and the material porosity adjusted in the simulation to correlate with the experimental results (see Supplementary Information).

Although there was minimal variation in media velocity through the scaffold, pressure decreased by approximately 450 Pa as it progressed (Figure 8B). Shear stress at the interface between the channels and the surface of the polyHIPE could not be plotted as flow is not tangential as it also goes through the polyHIPE material. Therefore, the simulation was repeated where the polyHIPE components were assumed to be non-porous to allow the shear stress to be approximated. At the base of the channels, this was approximately 0.8 to 1.4 Pa (Figure 8C). It is interesting to note that the shear stress experienced by the cells on the surface of the chip scales linearly with the flow, from 0.2–0.4 Pa at 0.8 mL/min to 0.8–1.4 Pa at 3.2 mL/min. The highest flow rate used in this study was 3.2 mL/min, which using CFD was calculated to correspond to a shear stress of 0.8 to 1.4 Pa. Were it possible to accurately model the shear stress at the interface of the porous material, these shear stress values would be slightly lower. This is because the velocity in the center of channel would be slightly lower than modeled as some flow would go through the polyHIPE pillars. Furthermore, the velocity at the interface of the porous pillars and channel is actually slightly greater than zero. Despite the actual shear stress being slightly lower than modeled, it remains in the range of estimated *in vivo* shear stress in bone of 0.8 to 3 Pa (Weinbaum et al., 1994; Rubin et al., 2006).

## Summary

In summary, organ/tissue-on-a-chip technologies have the potential to revolutionize pharmaceutical pre-clinical testing by increasing throughput whilst minimizing the financial and ethical concerns associated with *in vivo* evaluation. Since its inception, great efforts have gone into developing these devices for many organ types, but thus far bone has been comparatively overlooked. We have successfully created a microfluidic platform that contains the necessary 3D architecture and physiologically relevant FSS for an osteogenesis-on-a-chip device suitable for long-term culture. This has the potential to provide more accurate, cheaper and faster methods of investigating novel therapeutics for bone and sequestering of toxins into bone, in comparison to standard *in vitro* and *in vivo* testing.

## Experimental

Ethics approval was not required for this study.

### Materials

All chemicals were purchased from Sigma-Aldrich, United Kingdom unless otherwise stated.

### Bone Microfluidic Chip Fabrication – Bioreactor

To fabricate the bioreactor component of the bone microfluidic chip negative replica molding was utilized. The mold was created using stereolithography from polyethylene glycol diacrylate 700 g/mol (PEG-DA) made photocurable by incorporating a photoinitiator [diphenyl (2,4,6-trimethylbenzoyl)-phosphine oxide/2-hydroxy-2-methylpropiophenone, 50/50] at 4 wt%. To fabricate molds, glass slides were first cleaned using piranha solution (80% sulfuric acid: 20% hydrogen peroxide) for 3 h, washed with deionized water (diH_2_O), then placed in 10% MAPTMS [3-(trimethoxysilyl) propyl-methacrylate] in toluene (Fisher Chemical, United Kingodm) overnight at room temperature to add methacrylate groups to the surface. When required, slides were washed with methanol (Fisher Chemical, United Kingdom), and air dried. Photocurable PEG-DA was then added to the surface of the slide and structured into the mold using a picosecond PULSEAS P 355-300 laser. A 355 nm wavelength was separated by optical prism and focused through an objective lens with 10 × magnification (NA = 0.3) onto the glass slide-PEG-DA interface. A shutter was placed between the prism and the focusing lenses to allow beam modulation. Using a computer-aided design (CAD) application (A3200 movement composer, Aerotech, United Kingdom), and motorized stage capable of moving in all three axes [ANT130-XY for xy translation and PRO115 for z translation (both Aerotech, United Kingdom)], the focal spot of the laser was translated to produce the mold.

To enable easy pattern transfer and improve mold durability, an ultra-thin film of titanium (1.7 nm as determined by Quartz Crystal Microbalance) was coated on the surface via sputter coating. To create the bioreactor, the mold was used to stamp polydimethylsiloxane (PDMS, Sylgard 184, Dow Corning, United States). First, molds were cleaned using pressurized air, the silicone monomer and cross-linker agent were mixed (10:1) and the uncured PDMS poured onto the mold and left under vacuum for degassing. After eliminating gas bubbles, they were left for 2 h at 60°C to polymerize. The solid PDMS bioreactors were carefully peeled from the molds and the inlet and outlet created using a 2 mm biopsy punch. Finally, molds were cleaned with methanol. Due to the low surface energy of PDMS, the molds can be repeatedly reused.

### Bone Microfluidic Chip Fabrication – PolyHIPE Scaffold

To fabricate the polyHIPE scaffold component of the bone microfluidic chip, positive and subsequently negative replica molding was utilized. First, using the same protocol as the bioreactor component, a positive replica of the desired scaffold was produced from photocurable PEG-DA, then, a negative replica of this was created from PDMS. This negative replica was used to stamp the scaffolds from the polyHIPE material.

To create the polyHIPE, a HIPE composed of 70 wt% 2-ethylhexyl acrylate (EHA), 30 wt% isobornyl acrylate (IBOA), and porosity of 80% was prepared. To prepare the continuous phase of HIPE, the two monomers (EHA and IBOA), and a crosslinker (trimethylolpropane triacrylate, 18.28 wt% of the monomers) were mixed to form the organic component. A surfactant [Hypermer B246 (Croda Lubricants, United Kingdom)], and the photoinitiator were then added at 3 and 5 wt% of the organic mass, respectively. Once combined, the internal phase (deionized water, diH_2_O) was added drop-wise at a constant rate to the continuous phase whilst stirring at 350 rpm using a paddle stirrer (Pro40, SciQuip, United Kingdom) in a 100 mL beaker. Once added, the HIPE was stirred for a further 5 min. To prepare the polyHIPE scaffold, HIPE was poured into the PDMS negative replica of the scaffold and polymerized using a UV spot curer for 5 min at 100 W (Omnicure S1500, Excelitas Technologies). Once cured, it was peeled from the mold and washed in methanol three times before leaving overnight to dry.

### Bone Microfluidic Chip Fabrication – Device Assembly

Prior to device assembly, the polyHIPE scaffold was air plasma treated to overcome the inherent hydrophobicity of the material in order to promote cell attachment in accordance with the protocol of Owen et al. (2016). Briefly, scaffolds were placed on a platform wrapped with aluminum foil into the plasma chamber. The pressure inside the chamber was lowered to 0.18 mbar and the power set to 50 W to generate the plasma. Scaffolds were treated for 5 min.

To assemble the components, a glass slide was cleaned with acetone, then methanol, and finally diH_2_O. A plasma-treated polyHIPE scaffold was then inserted into the chamber of the bioreactor and placed into the plasma chamber face down along with the glass slide. The components were then plasma treated for 30 s at 50 W before gently placing the microfluidic chip onto the glass slide. The whole device was then left at 100°C for 10 s in an oven to complete the covalent sealing. A schematic of the device assembly process can be found in Figure 1.

### Scanning Electron Microscopy

All samples were mounted on a carbon tab, sputter coated with gold, then imaged using a Philips XL 30S FEG with an electron beam with an energy of 15 kV. Images were analyzed using the measurement tool in Image J.

### Cell Culture

Human embryonic stem cell-derived mesenchymal progenitors (hES-MPs) (Cellartis, Sweden) were used for all cell culture experiments. Cells were passaged in basal media [BM, minimum alpha medium (α-MEM, Lonza, United Kingdom], 10% fetal bovine serum (FBS, Labtech, United Kingdom), 2 mM L-glutamine, 100 μg/mL streptomycin and 100 U/mL penicillin), supplemented with 4 ng/mL human fibroblastic growth factor (Life Technologies, United Kingdom) in gelatine-coasted T75 flasks at 37°C, 5% CO_2_. Only cells between the 8th and 10th passage were used.

During experiments, hES-MPs were cultured in either OIM or supplemented media (SM). OIM is BM supplemented with ascorbic acid 2-phosphate (AA2P, 50 μg/mL, beta-glycerophosphate (βGP, 5 mM) and dexamethasone (100 nM). SM is the same composition as OIM but without dexamethasone.

To apply fluid flow to the chips, a commercial perfusion pump system (Ibidi, Germany) was used inside a standard incubator. To sterilize the chips, 70% ethanol was circulated through the chip for 2 h. Afterward, sterile PBS was flushed through the device three times, once per hour. Finally, to promote cell attachment, FBS was circulated for 12 h prior to seeding. To seed, 120,000 cells in 30 μL BM (∼110,000 cells/cm^2^) were injected through the inlet in the bioreactor and incubated for 1 h without flow to attach. Subsequently, either OIM or SM was perfused through the chips for up to 3 weeks with different unidirectional flow regimens. Flow was either continuous or intermittent. Continuous flow rates were either 0.8, 1.6, 2.4, or 3.2 mL/min, whilst the intermittent flow profile was 270 min of 0.8 mL/min followed by 90 min of 3.2 mL/min, repeated as a 6 h loop throughout the experiment. The required amount of the desired media was added to the pump’s syringes at the start of the experiment to cover the nutrient demand over time. Media was recirculated.

Cell death was observed after 24 h inside the sealed bone microfluidic chip when a flow rate of 0 mL/min was used (see Supplementary Information). Therefore, the static condition controls were seeded by adding a droplet of 120,000 cells in 30 μL of BM to the polyHIPE scaffold and leaving to attach for 1 h before adding 2 mL of either OIM or SM. Samples were maintained as an open culture within a well plate with media changes every 2–3 days.

### Resazurin Reduction Assay

Resazurin reduction (RR) assays were implemented to assess cell metabolic activity. 10 vol% resazurin solution (1 mM resazurin sodium salt in diH_2_O) was diluted in BM to make RR working solution. Scaffolds were removed from the chip and placed in well plates in 1 ml of the working solution, wrapped in aluminum foil and incubated for 4 h. Afterwards, 200 μl of the reduced solution was transferred to a 96 well plate in triplicate and read on a plate reader (Tecan infinite 200-pro) at λ*ex*: 540 nm, λ*em*: 590 nm.

### Cell Digestion

To produce lysates for ALP activity and DNA assays, scaffolds were removed from the chip, and added to 1 mL of cell digestion buffer (10 vol% cell assay buffer: 1.5M Tris-HCl, 1 mM ZnCl_2_, 1 mM MgCl_2_ in diH_2_O) in diH_2_O with 1 vol% Triton-X 100) in a well plate. They were then incubated for 30 min before being pulverized and the mixture transferred to a 1.5 mL microcentrifuge tube. Samples were refrigerated overnight, then exposed to a freeze-thaw cycle (−80°C, 10 min; 37°C, 15 min) three times, centrifuged for 5 min at 10,000 rpm and vortexed.

### Alkaline Phosphatase Activity

20 μL of cell lysate was combined with 180 μL of assay substrate [pNPP Substrate Kit (Thermo Fisher Scientific, United Kingdom)] in triplicate in a clear 96 well plate. The mixture was left at room temperature until a slight color change to yellow was observed or 30 min had passed to ensure measurements were taken during the linear phase of the reaction. Absorbance was then measured at 405 nm every minute for 30 min. ALP activity is expressed as nmol of pNP/minute, where one absorbance value equates to 19.75 nmol of pNP.

### DNA Quantification

Total DNA was quantified using a Quant-iT^®^ high sensitivity dsDNA assay kit (Thermo Fisher Scientific, United Kingdom) according to manufacturer instructions. Briefly, the Quanti-iT reagent was diluted 1:200 with the provided buffer to create a working solution. 90 μl of working solution was combined with 10 μL of lysate in triplicate in a black 96 well plate before shaking for 15 s and leaving to conjugate for 10 min. Fluorescence was then measured at λ*ex*: 485 nm, λ*em*: 535 nm and converted to ng of DNA per chip/culture well using a standard curve.

### Cell Fixation

Prior to calcium and collagen staining and microscopy, samples were fixed by removing the polyHIPE scaffold from the chip, washing twice with PBS and submerging in 3.7% formaldehyde for 30 min before rinsing a further two times in PBS. Samples were stored at 4°C in PBS until use.

### Calcium Deposition

Matrix calcium deposition by the cells was measured using Alizarin Red S (ARS) staining. Fixed samples were washed twice with diH_2_O, then 2 mL of ARS working solution (1 w/v% ARS dissolved in diH_2_O) was added to each scaffold and left for 30 min. ARS solution was removed, and samples were washed with diH_2_O with gentle orbital shaking until wash water remained clear. 5% perchloric acid was used to destain the samples for 15 min with gentle orbital shaking before transferring 150 μL of destain solution in triplicate to a clear 96 well plate and measuring absorbance at 405 nm. Absorbance was converted to concentration of ARS (μg/mL) using a standard curve.

### Collagen Synthesis

Total collagen content was detected using Direct Red 80 (DR80) staining. Samples were washed three times with water after performing the ARS assay and 2 mL of DR80 working solution (1 w/v% DR80 in saturated picric acid) was added to each sample before leaving for 12 h with orbital shaking at 100 rpm. After washing with diH_2_O, 0.2M sodium hydroxide:methanol (1:1) was added to destain the samples and left for 20 min with orbital shaking at 100 rpm. 150 μL of destain solution was transferred in triplicate to a clear 96 well plate and absorbance measured at 405 nm. The DR80 concentration was (μg/mL) calculated using a standard curve.

### Live Cell Tracking

For cell tracking experiments, hES-MPs were stained with CellTracker^TM^ Red CMTPX (Thermo Fisher Scientific, United Kingdom) prior to seeding according to manufacturer instructions. Briefly, the dye was dissolved in DMSO and diluted in α-MEM to a working concentration of 5 μM. After detaching with trypsin, cells were centrifuged and suspended in the working solution and incubated for 45 min. Cells were then recentrifuged and seeded as normal. Cells were imaged using fluorescence microscopy (Ti-E Nikon inverted microscope with a Nikon Intensilight CHGFI fluorescence unit, Nikon).

### Confocal Microscopy

A detailed protocol on the use of confocal microscopy for these polyHIPEs has been reported previously (Owen et al., 2016). Briefly, scaffolds with the highest resazurin reduction fluorescence were stained with DAPI (40-, 6-diamidino-2- phenylindole dihydrochloride) and Phalloidin-TRITC (Phalloidin–Tetramethylrhodamine B iso-thiocyanate) in order to view nuclei and f-actin, respectively. Confocal images (512 × 512 pixels) were obtained using an upright microscope (Axioskop 2 FS MOT Microscope, Carl Zeiss, Ltd., United Kingdom) with a 10 × objective (EC Plan-Neofluar 10 × /NA 0.30, Carl Zeiss, Ltd., United Kingdom) and a pixel dwell time of 2.56 μs. DAPI was detected using a tuneable Ti-Sapphire two-photon laser (λ_ex_: 800 nm, λ_em_: 435–485 nm) and Phalloidin-TRITC detected using a single photon laser (λ_ex_: 543 nm, λ_em_: 565–615 nm).

### Light Sheet Microscopy

On day 7 of culture samples were fixed and permeabilized in 0.5% Triton-X 100 in PBS for 10 min. Blocking with 5% goat serum was performed for 1 h at room temperature before the primary antibody [anti-acetylated α-tubulin clone 6-11B-1 produced in mouse (1 μg/ml)] was applied for 24 h at 5°C. The secondary antibody [goat anti-mouse IgG H&L Alexa Fluor^®^ 488 (2 μg/ml, Abcam, United Kingdom)] was then applied for 1 h at room temperature followed by counterstaining with DAPI. Three PBS washes were performed between each of the previous steps. The samples were imaged using a Z.1 light sheet microscope (Zeiss). Samples were mounted in 0.8% agarose (vol/vol in deionized water) within glass capillaries (size 4, Zeiss). Two 10 × NA 0.2 illumination optics (Zeiss) were used to illuminate the samples in combination with a W plan-apochromat 20 × /1.0 objective (Zeiss). Samples were excited using a 405 nm (20 mW) and a 488 nm (50 mW) laser. Z-stacks were taken and a maximum projection image created using the accompanied ZEN software.

### Histology

Fixed scaffolds were placed into cryosectioning molds and covered with optimal cutting temperature (OCT) compound (Leica). The molds were left under vacuum for 1 h to let the OCT permeate into the pores of the polyHIPE. Samples were then snap-frozen in a bath of liquid nitrogen before 8 μm sections were cut using a cryostat (Leica CM1860UV) and collected on slides. Slides were then stained with hematoxylin and eosin solution (H&E), using standard protocol for frozen samples. Slides were imaged using a light microscope (Motic).

### Computational Fluid Dynamics

Simulations of culture medium (modeled as water) flowing along the channels and through the porous polyHIPE were carried out at various volumetric flow rates (Q: 0.8, 1.6, 2.4, and 3.2 mL/min) which were assumed to be laminar with environmental pressure at the outlet fixed at 101325 Pa. A constant temperature of 37°C was assumed for all simulations using adiabatic walls with surface roughness from 0.5 to 100 μm. The geometry of all parts were generated in 3D including inlet and outlet tubes of diameter 2 mm (area 3.142 mm^2^). A spreadsheet table was generated to estimate values of velocity, Reynolds number, shear stress, and pressure drop. This spreadsheet used volumetric flow velocities on entrance and exit faces and approximate values along narrow channels of around h: 0.3 mm x w: 0.38 mm (area reduced to A: 0.114 mm^2^) with and estimated equivalent length from the 3D CAD L: 27 mm. Water was assumed to have a dynamic viscosity μ: 7.75 × 10^–4^ kg/(m.s) and density ρ: 1000 kg/m^3^. Model validation and simulation procedure is explained in detail at the Supplementary Information.

### Statistical Analysis

Statistical analysis was performed using GraphPad Prism (Version 7.00). To assess differences, either one-way or two-way ANOVA (depending on whether a response was affected by one or two factors) was used with Tukey’s or Sidak’s multiple comparison test, respectively, to evaluate the statistical significance. Differences were considered significant when *p* < 0.05 and notable significant differences are indicated on the figures or legends. All graphs are presented as mean ± standard deviation with the number of replicates stated in the figure legend.

## Data Availability Statement

The raw data supporting the conclusions of this article will be made available by the authors, without undue reservation.

## Author Contributions

HB, CP, GR, and FC: conceptualization and project administration. HB: data curation, formal analysis, and validation. CP, GR, and FC: funding acquisition, resources, and supervision. HB, RO, LB, and AG-G: investigation and methodology. HB and RO: visualization and writing – original draft. RO, GR, and FC: writing – review and editing. All authors contributed to the article and approved the submitted version.

## Conflict of Interest

CP was employed by company Eden Microfluidics. The remaining authors declare that the research was conducted in the absence of any commercial or financial relationships that could be construed as a potential conflict of interest.

## References

[B1] Aldemir Dikici B. Dikici S. Reilly G. C. Macneil S. Claeyssens F. (2019). A novel bilayer polycaprolactone membrane for guided bone regeneration: combining electrospinning and emulsion templating. *Materials* 12: 2643. 10.3390/ma12162643 31434207 PMC6721100

[B2] Aldemir Dikici B. Claeyssens F. (2020). Basic principles of emulsion templating and its use as an emerging manufacturing method of tissue engineering scaffolds. *Front. Bioeng. Biotechnol.* 8 1–32. 10.3389/fbioe.2020.00875 32903473 PMC7435020

[B3] Baker B. M. Chen C. S. (2012). Deconstructing the third dimension–how 3D culture microenvironments alter cellular cues. *J. Cell Sci.* 125 3015–3024. 10.1242/jcs.079509 22797912 PMC3434846

[B4] Barbetta A. Cameron N. R. (2004). Morphology and surface area of emulsion-derived (PolyHIPE) solid foams prepared with oil-phase soluble porogenic solvents: span 80 as surfactant. *Macromolecules* 37 3188–3201. 10.1021/ma0359436

[B5] Batra N. N. Li Y. J. Yellowley C. E. You L. Malone A. M. Kim C. H. (2005). Effects of short-term recovery periods on fluid-induced signaling in osteoblastic cells. *J. Biomech.* 38 1909–1917. 10.1016/j.jbiomech.2004.08.009 16023480

[B6] Bersini S. Jeon J. S. Dubini G. Arrigoni C. Chung S. Charest J. L. (2014). A microfluidic 3D in vitro model for specificity of breast cancer metastasis to bone. *Biomaterials* 35 2454–2461. 10.1016/j.biomaterials.2013.11.050 24388382 PMC3905838

[B7] Bose S. Roy M. Bandyopadhyay A. (2012). Recent advances in bone tissue engineering scaffolds. *Trends Biotechnol.* 30 546–554. 10.1016/j.tibtech.2012.07.005 22939815 PMC3448860

[B8] Brauchle E. Johannsen H. Nolan S. Thude S. Schenke-Layland K. (2013). Design and analysis of a squamous cell carcinoma in vitro model system. *Biomaterials* 34 7401–7407. 10.1016/j.biomaterials.2013.06.016 23827189

[B9] Cartmell S. H. Porter B. D. García A. J. Guldberg R. E. (2003). Effects of medium perfusion rate on cell-seeded three-dimensional bone constructs in vitro. *Tissue Eng.* 9 1197–1203. 10.1089/10763270360728107 14670107

[B10] Case N. Sen B. Thomas J. Styner M. Xie Z. Jacobs C. (2011). Steady and oscillatory fluid flows produce a similar osteogenic phenotype. *Calcif. Tissue Int.* 88 189–197. 10.1007/s00223-010-9448-y 21165611 PMC3588160

[B11] Chao P. Maguire T. Novik E. Cheng K.-C. Yarmush M. (2009). Evaluation of a microfluidic based cell culture platform with primary human hepatocytes for the prediction of hepatic clearance in human. *Biochem.Pharmacol.* 78 625–632. 10.1016/j.bcp.2009.05.013 19463793 PMC4487512

[B12] Delaine-Smith R. Macneil S. Reilly G. (2012). Matrix production and collagen structure are enhanced in two types of osteogenic progenitor cells by a simple fluid shear stress stimulus. *Eur. Cell Mater.* 24 162–174. 10.22203/ecm.v024a12 22865228

[B13] Delaine-Smith R. M. Reilly G. C. (2012). Mesenchymal stem cell responses to mechanical stimuli. *Muscles Ligaments Tendons J.* 2 169–180. 23738294 PMC3666521

[B14] Donahue S. W. Donahue H. J. Jacobs C. R. (2003). Osteoblastic cells have refractory periods for fluid-flow-induced intracellular calcium oscillations for short bouts of flow and display multiple low-magnitude oscillations during long-term flow. *J. Biomech.* 36 35–43. 10.1016/s0021-9290(02)00318-4 12485636

[B15] Du D. Furukawa K. S. Ushida T. (2009). 3D culture of osteoblast-like cells by unidirectional or oscillatory flow for bone tissue engineering. *Biotechnol. Bioeng.* 102 1670–1678. 10.1002/bit.22214 19160373

[B16] Esch M. B. Smith A. S. Prot J.-M. Oleaga C. Hickman J. J. Shuler M. L. (2014). How multi-organ microdevices can help foster drug development. *Adv. Drug Deli. Rev.* 69 158–169. 10.1016/j.addr.2013.12.003 24412641 PMC4095890

[B17] Esch M. B. Sung J. H. Yang J. Yu C. Yu J. March J. C. (2012). On chip porous polymer membranes for integration of gastrointestinal tract epithelium with microfluidic ‘body-on-a-chip’devices. *Biomed. Microdev.* 14 895–906. 10.1007/s10544-012-9669-0 22847474

[B18] Ferrell N. Ricci K. B. Groszek J. Marmerstein J. T. Fissell W. H. (2012). Albumin handling by renal tubular epithelial cells in a microfluidic bioreactor. *Biotechnol. Bioeng.* 109 797–803. 10.1002/bit.24339 22012446 PMC3285552

[B19] Fritton S. P. Weinbaum S. (2009). Fluid and solute transport in bone: flow-induced mechanotransduction. *Annu. Rev. Fluid Mech.* 41 347–374. 10.1146/annurev.fluid.010908.165136 20072666 PMC2805256

[B20] Genthial R. Beaurepaire E. Schanne-Klein M.-C. Peyrin F. Farlay D. Olivier C. (2017). Label-free imaging of bone multiscale porosity and interfaces using third-harmonic generation microscopy. *Sci. Rep.* 7:3419. 10.1038/s41598-017-03548-5 PMC5469828 28611441

[B21] Gong X. Fan Y. Zhang Y. Luo C. Duan X. Yang L. (2014). Inserted rest period resensitizes MC3T3-E1 cells to fluid shear stress in a time-dependent manner via F-actin-regulated mechanosensitive channel (s). *Biosci. Biotechnol. Biochem.* 78 565–573. 10.1080/09168451.2014.895657 25036951

[B22] Hadida M. Marchat D. (2019). Strategy for achieving standardized bone models. *Biotechnol. Bioeng.* 117 251–271. 10.1002/bit.27171 31531968 PMC6915912

[B23] Hao S. Ha L. Cheng G. Wan Y. Xia Y. Sosnoski D. M. (2018). A spontaneous 3D bone-on-a-chip for bone metastasis study of breast cancer cells. *Small* 14:e1702787. 10.1002/smll.201702787 29399951

[B24] Hinderer S. Schesny M. Bayrak A. Ibold B. Hampel M. Walles T. (2012). Engineering of fibrillar decorin matrices for a tissue-engineered trachea. *Biomaterials* 33 5259–5266. 10.1016/j.biomaterials.2012.03.075 22521489

[B25] Huh D. Hamilton G. A. Ingber D. E. (2011). From 3D cell culture to organs-on-chips. *Trends Cell Biol.* 21 745–754. 10.1016/j.tcb.2011.09.005 22033488 PMC4386065

[B26] Huh D. Leslie D. C. Matthews B. D. Fraser J. P. Jurek S. Hamilton G. A. (2012). A human disease model of drug toxicity–induced pulmonary edema in a lung-on-a-chip microdevice. *Sci. Transl. Med.* 4:159ra147. 10.1126/scitranslmed.3004249 23136042 PMC8265389

[B27] Jaasma M. J. O’brien F. J. (2008). Mechanical stimulation of osteoblasts using steady and dynamic fluid flow. *Tissue Eng. Part A* 14 1213–1223. 10.1089/ten.tea.2007.0321 18433309

[B28] Johnson D. L. Mcallister T. N. Frangos J. A. (1996). Fluid flow stimulates rapid and continuous release of nitric oxide in osteoblasts. *Am. J. Physiol. Endocrinol. Metab.* 271 E205–E208. 10.1152/ajpendo.1996.271.1.E205 8760099

[B29] Kimmins S. D. Cameron N. R. (2011). Functional porous polymers by emulsion templating: recent advances. *Adv. Funct. Mater.* 21 211–225. 10.1002/adfm.201001330

[B30] Knight E. Murray B. Carnachan R. Przyborski S. (2011). “Alvetex§: Polystyrene Scaffold Technology for Routine Three Dimensional Cell Culture,” in *3D Cell Culture: Methods and Protocols*, ed. Haycock J. W. (Totowa, NJ: Humana Press), 323–340. 10.1007/978-1-60761-984-0_20 21042981

[B31] Kreke M. R. Sharp L. A. Woo Lee Y. Goldstein A. S. (2008). Effect of intermittent shear stress on mechanotransductive signaling and osteoblastic differentiation of bone marrow stromal cells. *Tissue Eng. Part A* 14 529–537. 10.1089/tea.2007.0068 18352827

[B32] Lee A. Langford C. R. Rodriguez-Lorenzo L. M. Thissen H. Cameron N. R. (2017). Bioceramic nanocomposite thiol-acrylate polyHIPE scaffolds for enhanced osteoblastic cell culture in 3D. *Biomater. Sci.* 5 2035–2047. 10.1039/c7bm00292k 28726876

[B33] Li W. Gardinier J. D. Price C. Wang L. (2010). Does blood pressure enhance solute transport in the bone lacunar–canalicular system? *Bone* 47 353–359. 10.1016/j.bone.2010.05.005 20471508 PMC2902609

[B34] Malayeri A. Sherborne C. Paterson T. Mittar S. Asencio I. O. Hatton P. V. (2016). Osteosarcoma growth on trabecular bone mimicking structures manufactured via laser direct write. *Int. J. Bioprint.* 2 176–186.

[B35] Malfait A.-M. Little C. B. (2015). On the predictive utility of animal models of osteoarthritis. *Arthritis Res.Ther.* 17 225–225. 26364707 10.1186/s13075-015-0747-6 PMC4568581

[B36] Maschmeyer I. Lorenz A. K. Schimek K. Hasenberg T. Ramme A. P. Hübner J. (2015). A four-organ-chip for interconnected long-term co-culture of human intestine, liver, skin and kidney equivalents. *Lab Chip* 15 2688–2699. 10.1039/c5lc00392j 25996126

[B37] McGonigle P. Ruggeri B. (2014). Animal models of human disease: challenges in enabling translation. *Biochem. Pharmacol.* 87 162–171. 10.1016/j.bcp.2013.08.006 23954708

[B38] Melchels F. P. Feijen J. Grijpma D. W. (2010). A review on stereolithography and its applications in biomedical engineering. *Biomaterials* 31 6121–6130. 10.1016/j.biomaterials.2010.04.050 20478613

[B39] Michael Delaine-Smith R. Javaheri B. Helen Edwards J. Vazquez M. Rumney R. M. H. (2015). Preclinical models for in vitro mechanical loading of bone-derived cells. *BoneKEy Rep.* 4 728–728. 26331007 10.1038/bonekey.2015.97 PMC4549923

[B40] Mukhopadhyay R. (2007). *When PDMS isn’t the best.* Washington, DC: ACS Publications.

[B41] Neuži P. Giselbrecht S. Länge K. Huang T. J. Manz A. (2012). Revisiting lab-on-a-chip technology for drug discovery. *Nat. Rev. Drug Discov.* 11 620–632. 10.1038/nrd3799 22850786 PMC6493334

[B42] Novik E. Maguire T. J. Chao P. Cheng K. Yarmush M. L. (2010). A microfluidic hepatic coculture platform for cell-based drug metabolism studies. *Biochem. Pharmacol.* 79 1036–1044. 10.1016/j.bcp.2009.11.010 19925779 PMC3136813

[B43] Owen R. Bahmaee H. Claeyssens F. Reilly G. C. (2020a). Comparison of the anabolic effects of reported osteogenic compounds on human mesenchymal progenitor-derived osteoblasts. *Bioengineering* 7:12. 10.3390/bioengineering7010012 31972962 PMC7148480

[B44] Owen R. Sherborne C. Evans R. Reilly G. C. Claeyssens F. (2020b). Combined porogen leaching and emulsion templating to produce bone tissue engineering scaffolds. *Int. J. Bioprint.* 6 99–113. 10.18063/ijb.v6i2.265 PMC7415854 32782992

[B45] Owen R. Reilly G. C. (2018). In vitro models of bone remodelling and associated disorders. *Front. Bioeng. Biotechnol.* 6:134. 10.3389/fbioe.2018.00134 30364287 PMC6193121

[B46] Owen R. Sherborne C. Paterson T. Green N. H. Reilly G. C. Claeyssens F. (2016). Emulsion templated scaffolds with tunable mechanical properties for bone tissue engineering. *J. Mech. Behav. Biomed. Mater.* 54 159–172. 10.1016/j.jmbbm.2015.09.019 26458114 PMC4717122

[B47] Owen R. Sherborne C. Reilly G. C. Claeyssens F. (2015). Data for the analysis of PolyHIPE scaffolds with tunable mechanical properties for bone tissue engineering. *Data Brief* 5 616–620. 10.1016/j.dib.2015.09.051 26958618 PMC4773382

[B48] Paterson T. E. Gigliobianco G. Sherborne C. Green N. H. Dugan J. M. Macneil S. (2018). Porous microspheres support mesenchymal progenitor cell ingrowth and stimulate angiogenesis. *APL Bioeng.* 2:026103 10.1063/1.5008556 PMC6481713 31069300

[B49] Plunkett N. A. Partap S. O’brien F. J. (2009). Osteoblast response to rest periods during bioreactor culture of collagen–glycosaminoglycan scaffolds. *Tissue Eng. Part A* 16 943–951. 10.1089/ten.tea.2009.0345 19827912

[B50] Pusch J. Votteler M. Göhler S. Engl J. Hampel M. Walles H. (2011). The physiological performance of a three-dimensional model that mimics the microenvironment of the small intestine. *Biomaterials* 32 7469–7478. 10.1016/j.biomaterials.2011.06.035 21764120

[B51] Puwanun S. Delaine-Smith R. M. Colley H. E. Yates J. M. Macneil S. Reilly G. C. (2018). A simple rocker-induced mechanical stimulus upregulates mineralization by human osteoprogenitor cells in fibrous scaffolds. *J. Tissue Eng. Regen. Med.* 12 370–381. 10.1002/term.2462 28486747 PMC5836908

[B52] Robinson J. L. Mcenery M. A. P. Pearce H. Whitely M. E. Munoz-Pinto D. J. Hahn M. S. (2016). Osteoinductive PolyHIPE foams as injectable bone grafts. *Tissue Eng. Part A* 22 403–414. 10.1089/ten.tea.2015.0370 26739120 PMC4800270

[B53] Robling A. G. Burr D. B. Turner C. H. (2000). Partitioning a daily mechanical stimulus into discrete loading bouts improves the osteogenic response to loading. *J. Bone Miner. Res.* 15 1596–1602. 10.1359/jbmr.2000.15.8.1596 10934659

[B54] Robling A. G. Burr D. B. Turner C. H. (2001). Recovery periods restore mechanosensitivity to dynamically loaded bone. *J. Exp. Biol.* 204 3389–3399. 11606612 10.1242/jeb.204.19.3389

[B55] Robling A. G. Turner C. H. (2009). Mechanical signaling for bone modeling and remodeling. *Crit. Rev. Eukaryotic Gene Exp.* 19 319–338. 10.1615/critreveukargeneexpr.v19.i4.50 19817708 PMC3743123

[B56] Roseti L. Parisi V. Petretta M. Cavallo C. Desando G. Bartolotti I. (2017). Scaffolds for bone tissue engineering: state of the art and new perspectives. *Mater. Sci. Eng. C* 78 1246–1262. 10.1016/j.msec.2017.05.017 28575964

[B57] Rubin J. Rubin C. Jacobs C. R. (2006). Molecular pathways mediating mechanical signaling in bone. *Gene* 367 1–16. 10.1016/j.gene.2005.10.028 16361069 PMC3687520

[B58] Scheinpflug J. Pfeiffenberger M. Damerau A. Schwarz F. Textor M. Lang A. (2018). Journey into bone models: a review. *Genes* 9:247. 10.3390/genes9050247 29748516 PMC5977187

[B59] Schneider C. A. Rasband W. S. Eliceiri K. W. (2012). NIH Image to ImageJ: 25 years of image analysis. *Nat. Methods* 9 671–675. 10.1038/nmeth.2089 22930834 PMC5554542

[B60] Sherborne C. Owen R. Reilly G. C. Claeyssens F. (2018). Light-based additive manufacturing of PolyHIPEs: controlling the surface porosity for 3D cell culture applications. *Mater. Des.* 156 494–503. 10.1016/j.matdes.2018.06.061

[B61] Sieber S. Wirth L. Cavak N. Koenigsmark M. Marx U. Lauster R. (2018). Bone marrow-on-a-chip: long-term culture of human haematopoietic stem cells in a three-dimensional microfluidic environment. *J. Tissue Eng. Regen. Med.* 12 479–489. 10.1002/term.2507 28658717

[B62] Sikavitsas V. I. Bancroft G. N. Holtorf H. L. Jansen J. A. Mikos A. G. (2003). Mineralized matrix deposition by marrow stromal osteoblasts in 3D perfusion culture increases with increasing fluid shear forces. *Proc. Natl. Acad. Sci. U.S.A.* 100 14683–14688. 10.1073/pnas.2434367100 14657343 PMC299759

[B63] Srinivasan S. Ausk B. J. Poliachik S. L. Warner S. E. Richardson T. S. Gross T. S. (2007). Rest-inserted loading rapidly amplifies the response of bone to small increases in strain and load cycles. *J. Appl. Physiol.* 102 1945–1952. 10.1152/japplphysiol.00507.2006 17255366

[B64] Temiyasathit S. Jacobs C. R. (2010). Osteocyte primary cilium and its role in bone mechanotransduction. *Ann. N. Y. Acad. Sci.* 1192 422–428. 10.1111/j.1749-6632.2009.05243.x 20392268 PMC3999479

[B65] Torisawa Y.-S. Spina C. S. Mammoto T. Mammoto A. Weaver J. C. Tat T. (2014). Bone marrow–on–a–chip replicates hematopoietic niche physiology in vitro. *Nat. Methods* 11 663–669. 10.1038/nmeth.2938 24793454

[B66] Wagner I. Materne E.-M. Brincker S. Süßbier U. Frädrich C. Busek M. (2013). A dynamic multi-organ-chip for long-term cultivation and substance testing proven by 3D human liver and skin tissue co-culture. *Lab Chip* 13 3538–3547. 23648632 10.1039/c3lc50234a

[B67] Wang A.-J. Paterson T. Owen R. Sherborne C. Dugan J. Li J.-M. (2016). Photocurable high internal phase emulsions (HIPEs) containing hydroxyapatite for additive manufacture of tissue engineering scaffolds with multi-scale porosity. *Mater. Sci. Eng. C* 67 51–58. 10.1016/j.msec.2016.04.087 27287098

[B68] Wang L. Fritton S. P. Weinbaum S. Cowin S. C. (2003). On bone adaptation due to venous stasis. *J. Biomech.* 36 1439–1451. 10.1016/s0021-9290(03)00241-0 14499293 PMC3929109

[B69] Weinbaum S. Cowin S. Zeng Y. (1994). A model for the excitation of osteocytes by mechanical loading-induced bone fluid shear stresses. *J. Biomech.* 27 339–360. 10.1016/0021-9290(94)90010-8 8051194

[B70] Wu C. C. Li Y. S. Haga J. H. Wang N. Lian I. Y. Z. Su F. C. (2006). Roles of MAP kinases in the regulation of bone matrix gene expressions in human osteoblasts by oscillatory fluid flow. *J. Cell. Biochem.* 98 632–641. 10.1002/jcb.20697 16440309

[B71] Zhou J. Ellis A. V. Voelcker N. H. (2010). Recent developments in PDMS surface modification for microfluidic devices. *Electrophoresis* 31 2–16. 10.1002/elps.200900475 20039289

