# Supplementary material for: Design and Evaluation of an Osteogenesis-on-a-Chip Microfluidic Device Incorporating 3D Cell Culture
Source: Front Bioeng Biotechnol. 2020 Sep 8;8:557111. doi: 10.3389/fbioe.2020.557111 (PMC7509430; doi:10.3389/fbioe.2020.557111)
Supplement: Supplementary file 1 [file Data_Sheet_1.PDF]

Supplementary Material

1 Computational Fluid Dynamics

Simulations of culture medium (modelled as water) flowing along the channels and through the porous polyHIPE were carried out at various volumetric flow rates (Q: 0.8, 1.6, 2.4 and 3.2 mL/min) which were assumed to be laminar with environmental pressure at the outlet fixed at 101325 Pa. A constant temperature of 37℃ was assumed for all simulations using adiabatic walls with surface roughness from 0.5 µm to 100 µm. The geometry of all parts was generated in 3D including inlet and outlet tubes of diameter 2 mm (area 3.142mm^2). A spreadsheet table was generated to estimate values of velocity, Reynolds number, shear stress and pressure drop. This spreadsheet used volumetric flow velocities on entrance and exit faces and approximate values along narrow channels of around *h*: 0.3 mm x *w*: 0.38 mm (area reduced to *A*: 0.114 mm^2) with an estimated equivalent length from the 3D CAD *L*: 27 mm. Water was assumed to have a dynamic viscosity µ: 7.75e-4 kg/(m.s) and density ρ: 1000 kg/m^3.

Assuming the flow only goes over the polyHIPE material, Reynolds number was estimated for the maximum flow and the minimum height as follows assuming six hexagonal channels with the same flow rate:

$$Re = \frac{\rho v h}{\mu} = \frac{\rho Q}{w\mu} = \frac{1000\times(3.2\times1e-6/60)}{6\times0.0003\times7.75e-4} = 38.23 \qquad \text{(Eq.1)}$$

This theoretical calculation demonstrates that flow can be assumed as laminar in simulations as it is well below the *Re* = 2000 transition from laminar to turbulent flow.

The velocities are estimated at three different locations: inlet, three straight channels and six narrow hexagonal channels.

$$v_i = \frac{Q}{A} = \frac{(3.2\times1e-6/60)}{\pi\times0.001^2} = 0.01697 \text{ m/s}$$

$$v_s = \frac{Q}{A} = \frac{(3.2\times1e-6/60)}{3\times0.00042\times0.0005} = 0.08422 \text{ m/s}$$

$$v_h = \frac{Q}{A} = \frac{(3.2\times1e-6/60)}{6\times0.0003\times0.00038} = 0.07794 \text{ m/s} \qquad \text{(Eq.2)}$$

The theoretical values obtained for velocities represent the average velocity in the channel and are multiplied by 2 in order to obtain the maximum velocity in the middle of the channel ($v_{max} = 0.168\ m/s$). Velocity is assumed to be zero at the walls as part of the no-slip boundary condition.

Theoretical approximate values of shear stress were also calculated prior to simulation as follows:

$$\tau = \frac{6v\mu}{h} = \frac{6Q\mu}{6wh^2} = \frac{6\times(3.2\times1e-6/60)\times7.75e-4}{6\times0.0003\times0.00038^2} = 0.9541 \text{ Pa} \qquad \text{(Eq.3)}$$

Finally, the pressure drop along the length of the bone microfluidic chip was calculated using the Darcy friction factor:

$$\Delta P_f = \frac{kL\rho v^2}{Re2h} = \frac{kL\rho Q^2}{Re2w^2h^36^2} = \frac{57\times0.027\times1000\times(3.2\times1e{-6}/60)^2}{38.23\times2\times0.0003^2\times0.00038^3\times6^2} = 322 \text{ Pa} \qquad \text{(Eq.4)}$$

Where k: shape factor (64 for circular pipe, 57 for square pipe), ρ: density, *v*: velocity, *h*: height, μ: dynamic viscosity, *Q*: flow rate, *w*: width.

3D simulations were then performed using the material properties and boundary conditions above in order to compare results to the calculated values. The initial simulations assumed that the polyHIPE material is non-porous and that the water flows along the channels. The velocities shown in Supplementary Fig. 1A are very close to the theoretical values of 0.168 m/s in the centre of the channel. These velocities were quite similar in all hexagonal channels in flow (X) direction. The pressure drop shown in supplementary Fig. 1B, 101662-101325=337 Pa, was close to the theoretical value of 322 Pa. The shear stresses shown in supplementary Fig. 1C&1D are in range of 1.5 Pa in the centre of the channel instead of 0.95 Pa estimated with simplifications.

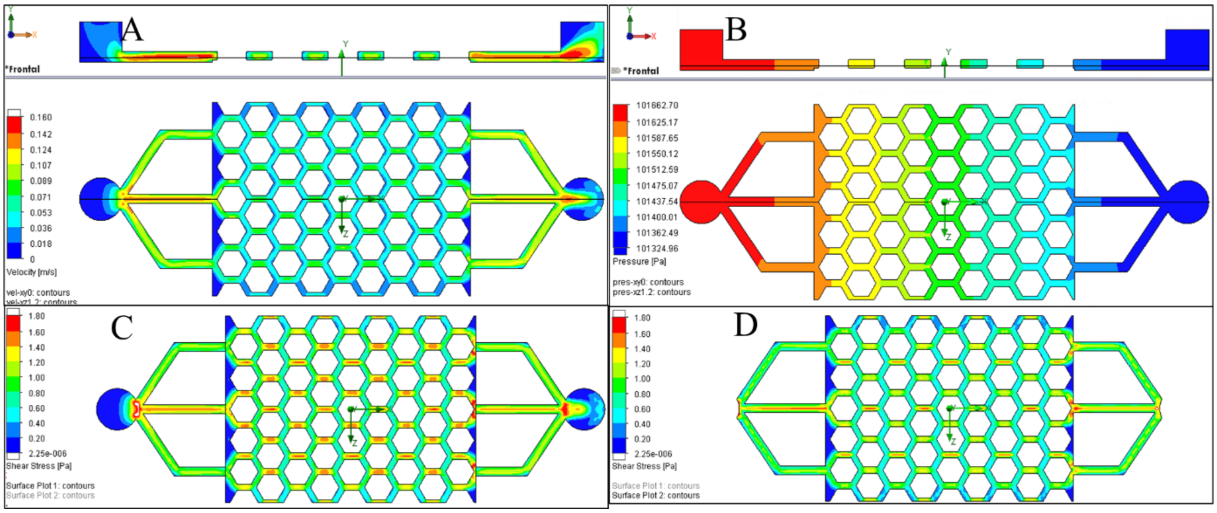

Supplementary Figure 1: Simulation results for non-porous polyHIPE at Q: 3.2 mL/min with A) velocities on mid-section, B) pressure drop on mid-section and C) shear stress on bottom polyHIPE surface and D) top PDMS surface.

Once the model was validated for a non-porous polyHIPE, we expanded it to permit the use of porous polyHIPEs. For this, a second validation was required to understand how water flows through the polyHIPE. A pressure drop device was manufactured to fit disks of polyHIPE of different thickness to allow a circular section of diameter 10.5 mm. This setup is shown in supplementary Fig. 2A. Water flow was modulated by means of a pump to obtain velocities in the range of 0 to 0.012 m/s. The pressure drop was measured using a differential pressure meter PCE-917 with a range of ±7000 mbar (±700000 Pa) and precision ±5 mbar (±500 Pa) or ±0.05 psi (±344.7 Pa). With these tolerances it was found that measurements were readable for the range of velocities of this study with a minimum thickness of 5 mm. A curve fit of experimental results (supplementary Fig. 2B) with an acceptable regression coefficient (R^2=0.945) showed a linear dependency of pressure drop for each mm that the flow goes into the polyHIPE with velocity as follows:

$$\Delta P_p = stv = st\frac{Q}{A} = 47761 \times 5 \times 0.0006 = 143.3 \text{ Pa} \qquad \text{(Eq.5)}$$

Where s: slope of curve fit, v: velocity, t: thickness of polyHIPE, Q: flow rate, A: cross section.

A model of the experiment was prepared to evaluate the pressure drop. The simulation predicted a pressure drop of 101456-101325=131 Pa (supplementary Fig. 2C) instead of the 143.3 Pa calculated theoretically for a constant speed of 0.0006 m/s. Simulation results for velocities found a deviation in shape of velocities from the parabolic around the porous polyHIPE (supplementary Fig. 2D). In a parabolic profile the velocities in the centre should be 2×0.0006=0.0012 m/s.

Once the simulation of flow through the polyHIPE was calibrated, the simulation could be repeated with the scaffold assumed to be porous. For a flow rate of 3.2 mL/min, it was observed in the simulation that water was flowing through bulk polyHIPE material but with very low speed (supplementary Fig. 3A). Surprisingly, the presence of the porous polyHIPE changed the velocity profile, obtaining slightly higher velocities in other regions. The inlet pressure also increased from 101666 to 101784 Pa (ΔP from 337 to 450 Pa, supplementary Fig. 3B). The shear stress also increased to around 1.8 Pa, slightly higher than the 1.6 Pa observed with non-porous polyHIPE due to higher velocities on entrance channels. It is not possible to plot shear stress on the polyHIPE surface as flow is not tangential as it also goes through the polyHIPE (supplementary Fig. 3C). On the PDMS surface shear stress increases up to 2.17 Pa due to the change in velocity profile due to the vicinity of porous polyHIPE. This is observed in supplementary Fig. 3D, where channels seem to be narrower than the non-porous configuration.

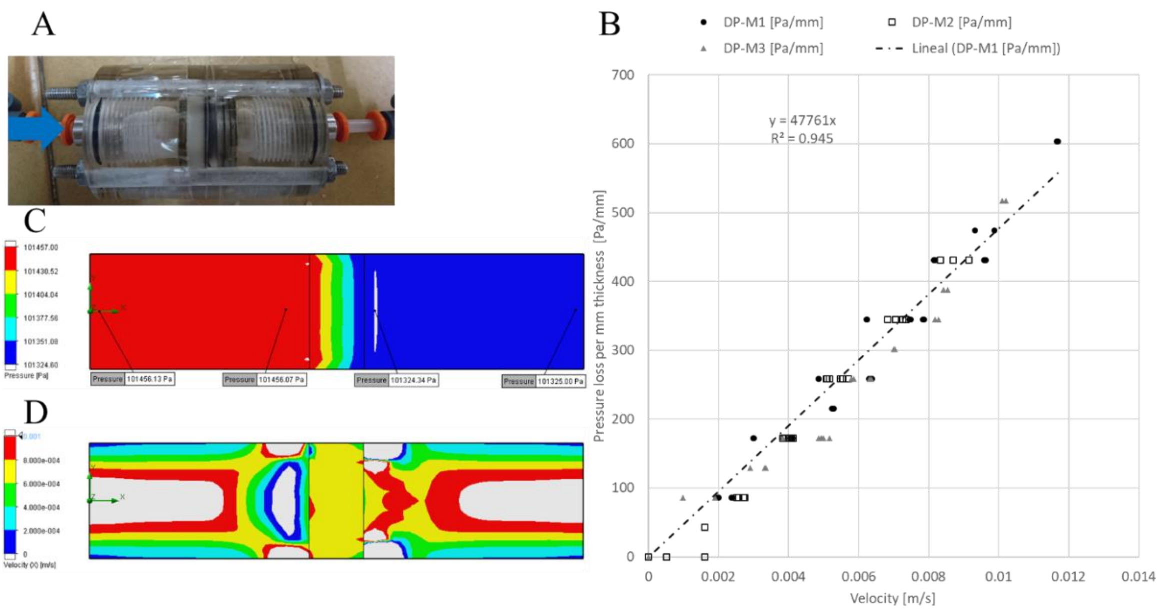

Supplementary Figure 2: Pressure drop across polyHIPE. (A) experimental setup, (B) experimental results and curve fit, (C) simulation results of pressure drop and (D) simulation velocities.

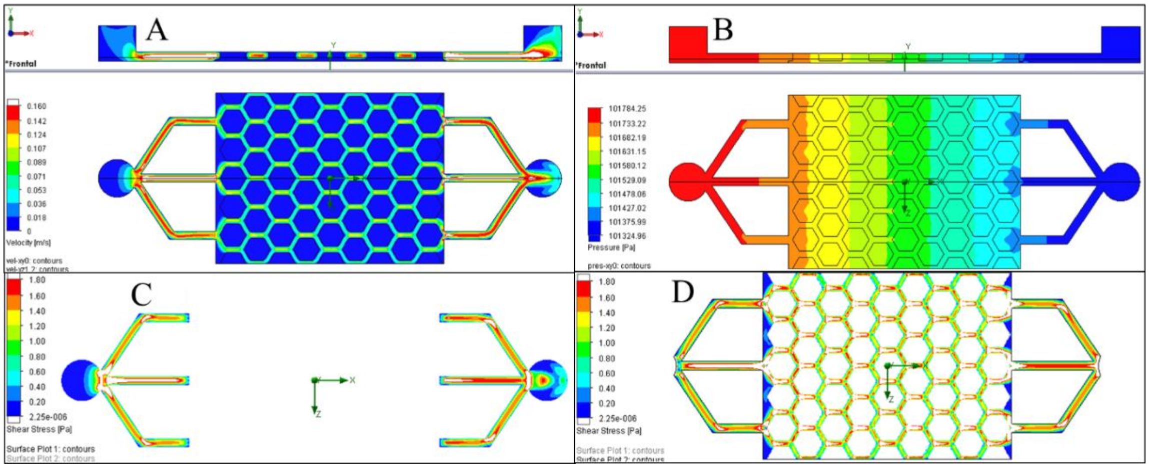

Supplementary Figure 3: Simulation results for porous polyHIPE at Q: 3.2ml/min with (A) velocities on sectioned part, (B) pressure drop on sectioned part and shear stress on (C) bottom polyHIPE surface and (D) top PDMS surface.

All simulations were repeated for the lower flow rates (Q: 0.8, 1.6, 2.4 mL/min), showing in all cases laminar flow, and therefore all values of Reynolds, velocity, shear stress and pressure drop, were linear with flow rate. This is in agreement with linearity shown through equations 1 to 5 and shown in supplementary Fig. 4.

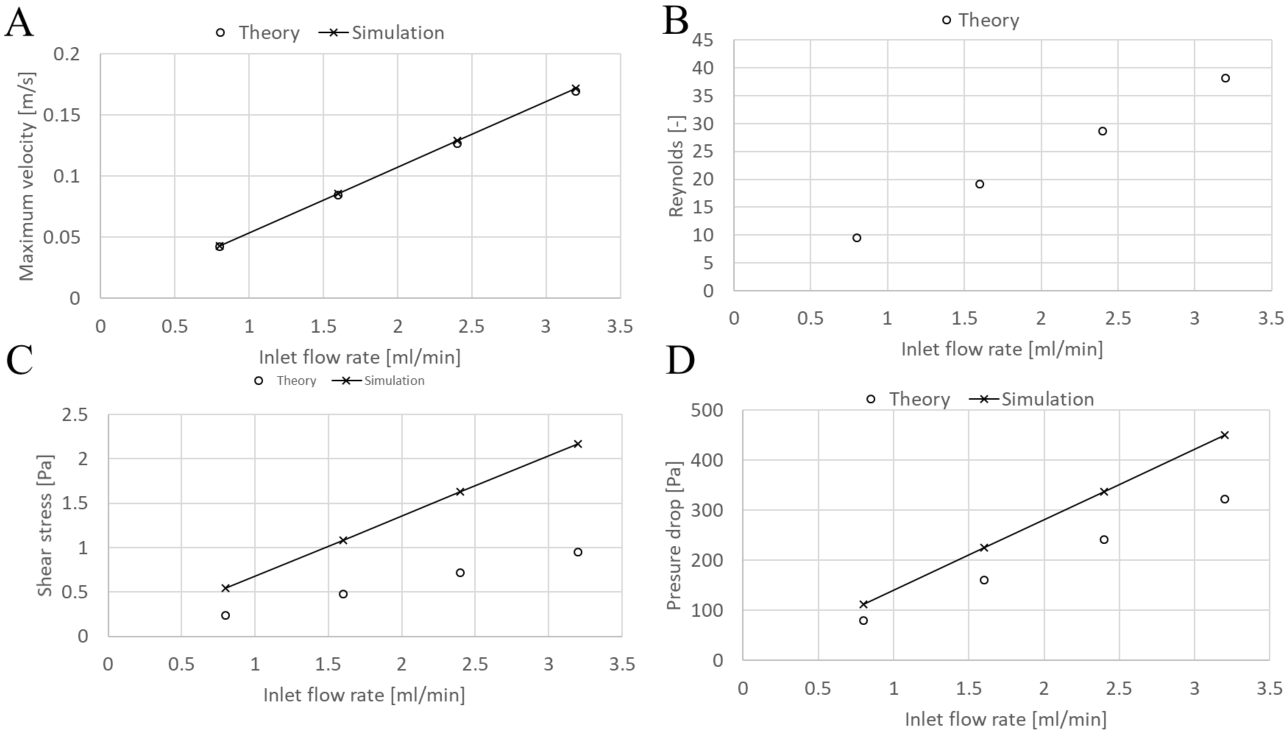

Supplementary Figure 4: Theoretical and simulation values for different flow rates for (A) maximum velocity, (B) Reynolds (only theory), (C) maximum shear stress and (D) pressure drop.

2 Static conditions within a sealed bone microfluidic chip results in cell death

The metabolic activity of hES-MPs after 24 hours was compared in sealed chips at 0 mL/min (static) and 0.8 mL/min (the lowest flow rate achievable). In static chips, metabolic activity was significantly lower, indicating cell death (Supplementary Fig. 5). Therefore, static controls were performed in open culture.

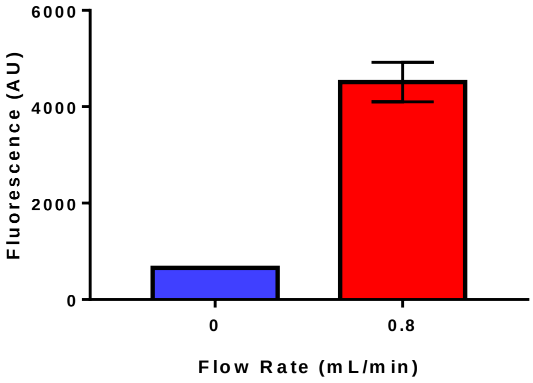

Supplementary Figure 5: Metabolic activity after 24h of hES-MPs cultured in sealed bone microfluidic chips in static and low-flow conditions
